# Caffeine Impairs Myocardial Blood Flow Response to Physical Exercise in Patients with Coronary Artery Disease as well as in Age-Matched Controls

**DOI:** 10.1371/journal.pone.0005665

**Published:** 2009-05-22

**Authors:** Mehdi Namdar, Tiziano Schepis, Pascal Koepfli, Oliver Gaemperli, Patrick T. Siegrist, Renate Grathwohl, Ines Valenta, Raphael Delaloye, Michael Klainguti, Christophe A. Wyss, Thomas F. Lüscher, Philipp A. Kaufmann

**Affiliations:** 1 Cardiac Imaging, University Hospital, Zurich, Switzerland; 2 Zurich Center for Integrative Human Physiology (ZIHP), University of Zurich, Zurich, Switzerland; University of British Columbia, Canada

## Abstract

**Background:**

Caffeine is one of the most widely consumed pharmacologically active substances. Its acute effect on myocardial blood flow is widely unknown. Our aim was to assess the acute effect of caffeine in a dose corresponding to two cups of coffee on myocardial blood flow (MBF) in coronary artery disease (CAD).

**Methodology/Principal Findings:**

MBF was measured with ^15^O-labelled H2O and Positron Emission Tomography (PET) at rest and after supine bicycle exercise in controls (*n* = 15, mean age 58±13 years) and in CAD patients (*n* = 15, mean age 61±9 years). In the latter, regional MBF was assessed in segments subtended by stenotic and remote coronary arteries. All measurements were repeated fifty minutes after oral caffeine ingestion (200 mg). Myocardial perfusion reserve (MPR) was calculated as ratio of MBF during bicycle stress divided by MBF at rest. Resting MBF was not affected by caffeine in both groups. Exercise-induced MBF response decreased significantly after caffeine in controls (2.26±0.56 vs. 2.02±0.56, P<0.005), remote (2.40±0.70 vs. 1.78±0.46, P<0.001) and in stenotic segments (1.90±0.41 vs. 1.38±0.30, P<0.001). Caffeine decreased MPR significantly by 14% in controls (P<0.05 vs. baseline). In CAD patients MPR decreased by 18% (P<0.05 vs. baseline) in remote and by 25% in stenotic segments (P<0.01 vs. baseline).

**Conclusions:**

We conclude that caffeine impairs exercise-induced hyperaemic MBF response in patients with CAD to a greater degree than age-matched controls.

## Introduction

Caffeine is a xanthine derivative and one of the most widely consumed pharmacologically active substances. As caffeine consumption has been linked to a potential adverse cardiovascular outcome the effects of coffee drinking on heart disease have been extensively studied in the past decades. The existing outcome data on the long-term effect of caffeine consumption on human health is inconsistent and therefore subject of an ongoing controversy [1]. The inconsistency of the literature may in part be due to differences in study design or the long time between the assessment of coffee intake and the occurrence of cardiovascular events in other reports [2]. Even less is known about the acute impact of caffeine on MBF, although caffeine and related naturally occurring xanthine derivatives are estimated to be consumed by 80% of the adult population in western countries [3]. While one follow-up study found a beneficial effect of coffee [4], some but not all prospective and epidemiological studies have shown an association of coffee drinking with increased cardiovascular morbidity and mortality [1], [5]–[8] with some results supporting a dose-dependent risk of a cardiovascular event [5]. A recent study with a case crossover design suggests that transient exposure to coffee might represent a trigger of a first nonfatal myocardial infarction [6]. Similarly, the role of coffee in the development of cardiovascular risk factors is controversial, as it has been linked to both elevated [7] and reduced [8] but also to unchanged blood pressure [9]. In one interesting study, coffee as well as caffeine alone induced comparable increases in sympathetic nerve activity and blood pressure in non-habitual coffee drinkers, whereas habitual coffee drinkers exhibited lack of blood pressure increase despite sympathetic nerve activation to coffee suggesting that ingredients other than caffeine may be responsible for cardiovascular activation [14]. Dietary intake of caffeine may also stimulate local and/or systemic catecholamine release causing an alpha-2-receptor-mediated coronary vasomotion [10]. In addition, caffeine might directly alter the vasomotor tone by antagonizing A1, A2A and A2B adenosine receptors on smooth muscle cells of the vessel wall and herewith reduce adenosine-mediated vasodilatation [16], [17] resulting in a decreased myocardial blood flow (MBF) response to exercise stress [18]–[20]. In fact, we recently showed that oral intake of caffeine at a dose corresponding to two cups of coffee significantly decreases bicycle exercise-induced hyperaemic MBF response in healthy volunteers [11] suggesting that hyperaemic response to exercise may at least in part depend on intrinsic adenosine production [12], [13]. Interestingly, the adverse effect of caffeine on hyperaemic response to exercise was even more pronounced during exposure to hypoxia [11], a situation mimicking the oxygen deprivation found in coronary artery disease (CAD) patients in whom compromised compensatory mechanisms do not allow adequate MBF increase to match the exercise-induced increase in oxygen consumption [14]. As adenosine production is linked to myocardial pO_2_ [15] and the major stimulus for its production seems to be an imbalance between oxygen delivery and oxygen demand we studied the hypothesis that caffeine consumption impairs the hyperaemic MBF response to exercise in CAD patients.

## Results

MBF was assessed using positron emission tomography (PET) at rest and during bicycle exercise at baseline and after oral administration of caffeine 200 mg. At baseline, caffeine level was 5.8±5.8 µmol/L in controls and increased fifty minutes after caffeine ingestion to 27.7±9.3 µmol/L (P<0.001 vs. baseline). In CAD patients caffeine level increased from 2.1±3.5 µmol/L to 20.6±9.9 µmol/L (P<0.001 vs. baseline). In both groups heart rate, blood pressure, and rate-pressure product (RPP; systolic blood pressure x heart rate) were not affected by caffeine neither at rest nor at peak exercise (Table 1). Comparable values for absolute and percent predicted workload was achieved by controls (95±30 Watts; 101±20%) and CAD patients (106±26 Watts; 97±11%, P = ns vs. controls) in the supine position within the scanner.

**Table 1:** Hemodynamics.

Age-matched controls (n = 15)				CAD Patients (n = 15)		
***Rest***	**Baseline**	**Caffeine**	**P**	**Baseline**	**Caffeine**	**P**
SBP (mmHg)	131±160	133±180	ns	132±140	133±170	ns
DBP (mmHg)	78±11	81±90	ns	76±13	77±10	ns
MAP (mmHg)	96±11	98±10	ns	95±12	96±10	ns
HR (bpm)	68±11	67±13	ns	65±11	64±11	ns
RPP (mmHg×bpm)	8870±1256	8807±1280	ns	8589±1991	8551±1670	ns
***Peak Exercise***	**Baseline**	**Caffeine**	**P**	**Baseline**	**Caffeine**	**P**
SBP (mmHg)	167±240	173±210	ns	166±190	173±170	ns
DBP (mmHg)	97±13	100±130	ns	93±15	98±14	ns
MAP (mmHg)	118±130	124±130	ns	118±140	123±130	ns
HR (bpm)	118±190	116±250	ns	113±170	113±190	ns
RPP (mmHg×bpm)	19515±36020	20105±47990	ns	18814±42380	19459±38260	ns

DBP = diastolic blood pressure (BP); HR = heart rate; MAP = mean BP; RPP = rate pressure product; SBP = systolic BP.

P-values are given for the comparison of baseline vs. caffeine.

**Figure 1:**
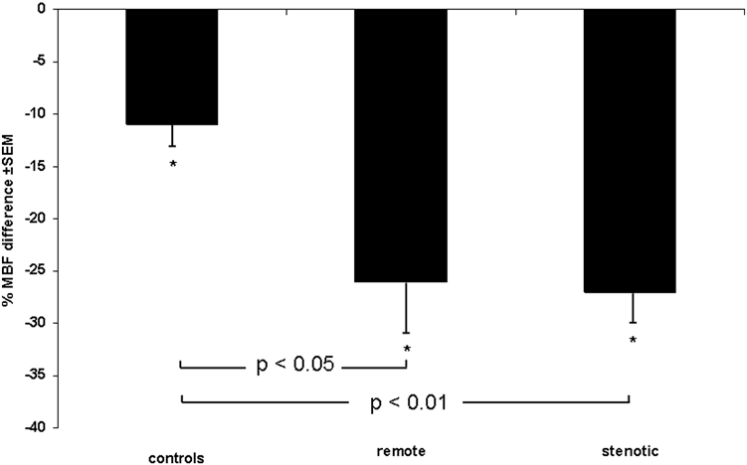
Exercise-induced hyperemia: effects of caffeine. Caffeine decreases exercise-induced hyperaemic MBF. This effect was most prominent in stenotic segments of CAD patients. *P<0.005 for the comparison versus baseline.

**Figure 2:**
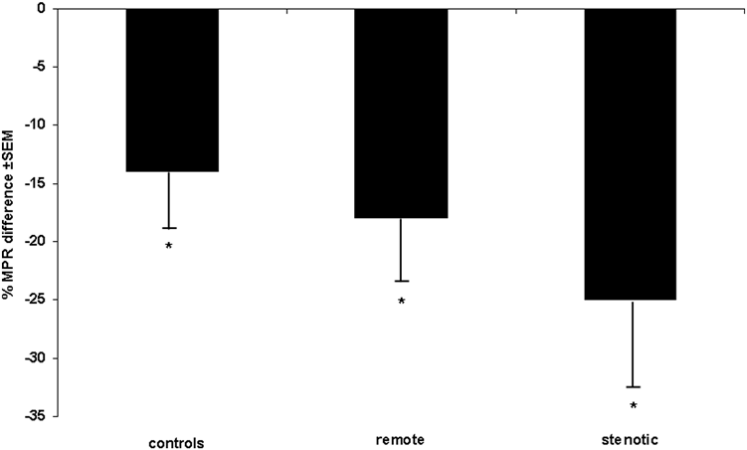
Myocardial perfusion reserve: effects of caffeine. Caffeine decreases myocardial perfusion reserve. This effect was most pronounced in stenotic segments of CAD patients. *P<0.05 for the comparison versus baseline.

**Figure 3:**
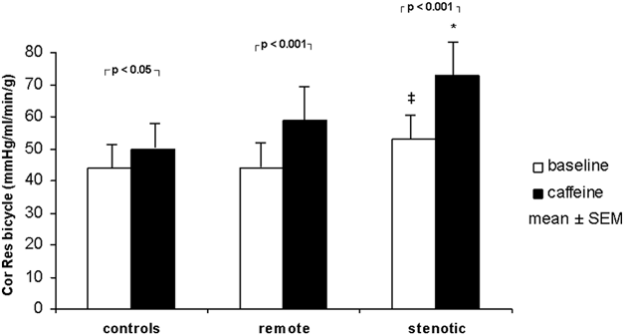
Coronary resistance during physical exercise. Coronary resistance at exercise increased in all groups. A massive increase in resistance was found in stenotic segments. ‡*P*<0.05 for the comparison versus remote. *P<0.05 for the comparison versus remote segments and age-matched controls.

In the control group mean global resting MBF remained unchanged after caffeine ingestion (1.21 vs. 1.26 mL/min/g, P = ns), while bicycle exercise-induced hyperaemic MBF decreased significantly by 11% (2.26±0.56 vs. 2.02±0.56 mL/min/g, P<0.005) as reflected by a significant increase in coronary resistance (+14%, P<0.05). This resulted in a significant decrease in exercise MPR (−14%, P<0.05) (Table 2). MBF in stenotic segments of CAD patients (0.94±0.26 mL/min/g) was lower than in remote segments (1.1±0.23 mL/min/g, P<0.05) and lower than global MBF in age-matched controls (P<0.05). Caffeine had no significant impact on MBF at rest neither in remote (−9%, P = ns) nor in stenotic segments (−3%, P = ns). Exercise-induced hyperaemic MBF was significantly lower in stenotic (1.90±0.41 mL/min/g) compared to remote segments (2.40±0.70 mL/min/g, P<0.05) and compared to controls (P<0.05) before caffeine (baseline) and decreased significantly after caffeine by 26% in remote (P<0.001 vs. baseline) and by 27% in stenotic segments (P<0.001 vs. baseline). Thus, the inhibitory effect of caffeine on MBF response to exercise was significantly less pronounced in controls than in CAD patients (P<0.05) (Figure 1) in whom MPR was significantly affected by caffeine with a decrease by 18% in remote (P<0.05) and by 25% in stenotic segments (P<0.01) (Figure 2). Accordingly, caffeine caused a significant increase in coronary resistance during bicycle exercise by 34% (P<0.001) in remote and by 38% in stenotic segments (P<0.001) (Figure 3).

**Table 2:** Myocardial Blood Flow, Coronary Resistance and Flow Reserve.

	Age-matched controls (n = 15)						CAD patients (n = 15)		
				remote segments			stenotic segments		
	Baseline	Caffeine	P	Baseline	Caffeine	P	Baseline	Caffeine	P
MBF rest	1.21±0.32	1.26±0.32	ns	1.1±0.23	1.0±0.26	ns	0.94±0.26* †	0.91±0.24†	ns
MBF ex	2.26±0.56	2.02±0.56	<0.005	2.4±0.7	1.78±0.46	<0.001	1.9±0.41*	1.38±0.3* †	<0.001
Cor Res rest	85±27	82±20	ns	89±17	102±32	ns	108±31* †	116±52†	ns
Cor Rest ex	44±13	50±17	<0.05	44±17	59±20	<0.001	53±17*	73±20* †	<0.001
MPR	1.94±0.61	1.67±0.59	<0.05	2.24±0.73	1.84±0.54	<0.05	2.1±0.53	1.58±0.38	<0.01

MBF, myocardial blood flow (ml/min/g). Cor Res, coronary resistance (mmHg/ml/min/g). MPR, myocardial perfusion reserve (relative values). Ex, exercise.

* P<0.05 for the comparison with remote segments.

† P<0.05 for the comparison with age-matched controls.

## Discussion

### Reproducibility and Generalizability of the findings

Our data provide evidence that caffeine consumption at a dose corresponding to two cups of coffee significantly affects myocardial blood flow in humans. This is line with the results of former studies, suggesting that the very effect of caffeine on myocardial blood flow is reproducible. Indeed, we showed in a recent study that oral intake of caffeine at the same dose as in the present study significantly decreases bicycle exercise-induced myocardial hyperaemic flow response in young healthy volunteers, particularly during hypoxia [11]. The effect of caffeine in the present study was most pronounced during physical exercise in CAD patients resulting in a significant decrease in myocardial perfusion reserve. The largest decrease in exercise-induced hyperaemic MBF by caffeine was found in myocardial regions subtended by stenotic coronary arteries of CAD patients. Accordingly, caffeine decreased MPR in CAD patients predominantly in the same segments. This is reflected by a marked increase in coronary resistance at exercise in these segments without any change in systemic hemodynamic conditions. Based upon our results and the given evidence of former studies, the microcirculatory changes due to the direct effect of caffeine can be considered as real as well as reproducible.

### Mechanisms supporting the findings and clinical implications

The present study supports that exercise-induced hyperaemic MBF response may at least in part be mediated by intrinsic adenosine and provides evidence that CAD patients are more susceptible to the antagonizing effects of caffeine than controls as their hyperaemic MBF response to exercise was significantly more impaired. Accordingly, previous reports in experimental animals, where an increase in MBF after aminophylline infusion has been attributed to a stimulation of alpha-1-adrenoreceptors when adenosine release is not prominent while during situations with increased adenosine release the inhibitory effect of aminophylline on adenosine receptors predominated over the beneficial effects of alpha-1-adrenoreceptor stimulation [23]. The finding of significantly decreased MPR after caffeine consumption seems not to have a direct and clinically evident negative impact in daily life in healthy individuals, indicating that a MPR of about 1.67 is largely sufficient. In stenotic segments of CAD patients, however, the MPR decrease was significantly more pronounced than in remote segments. This may raise questions about the safety of caffeine in patients with yet impaired MPR. Importantly, measured serum caffeine levels after ingestion of 200 mg of caffeine are comparable with serum caffeine levels observed after drinking two cups of coffee or a triple espresso [24], suggesting that serum concentrations measured and reported in our study are likely to occur with common ingestion of caffeine containing beverages. Several mechanisms may be involved in the differential effects of caffeine on exercise MBF in CAD versus controls. First, adenosine acts as an endogenous determinant of ischemic tolerance [25] and adenosinergic cardioprotection plays a key role in ischemic preconditioning. Therefore, ischemic segments may be more susceptible to antagonizing effects of caffeine. Second, the concentration-response relationship for interstitial adenosine-induced coronary vasodilatation is reported to be steep with a large receptor reserve for adenosine to cause an increase in MBF [26]. Receptor reserve is a pharmacological term for the phenomenon wherein the increment of functional response caused by an increase of agonist concentration is proportionately greater than the increment of receptor occupancy [26]. Studies with both agonists and antagonists indicate substantial yet submaximal receptor activation by endogenous adenosine during ischemia [27] supporting the possibility of reduced receptor reserve in ischemic myocardium. This may also contribute to the enhanced caffeine effect in ischemic segments. We have used bicycle exercise stress for stimulation of hyperaemic MBF as a physiological stimulus reflecting the natural response of the coronary arteries while a pharmacologic stimulus alone may have been of limited value in the assessment of the pathophysiological changes [20], [24], [26].

### Limitations and strengths of the study

A limitation of the present study is that data acquisition was obtained in the immediate post-exercise period when the cardiac power output is considerably decreased and when flow is also expected to fall rapidly. Therefore, we assess the impact of caffeine on an MBF value representing the average value over 5 minutes of recovery, rather than the peak MBF value at maximal exercise. Similarly, MPR during exercise might have been slightly higher than the value assessed post exercise. In addition, no placebo-controlled study design was followed. However, the fact that every subject was used as its own control strengthens our results and the validity of this method has been recently documented in a repeatability study [17]. For safety reasons the study did not allow inclusion of CAD patients with high-grade stenoses. As a result, our study was not designed to detect ischemia by ST-segment depression. In summary, our results show that exercise-induced hyperaemic MBF response can be blunted by caffeine. This may jeopardize patients with CAD.

### Conclusion

We conclude that caffeine impairs exercise-induced hyperaemic MBF response in patients with CAD to a greater degree than age-matched controls.

## Materials and Methods

### Study population

We studied 15 CAD patients (mean age 61±9 years, 13 males, 2 females) with angiographically documented single- or two-vessel disease, with a luminal stenosis of more than 50% in at least one epicardial artery. All patients had stable CAD in the Canadian Cardiovascular Society (CCS) functional class II to III. Only patients with mild (>50% to 70%) or moderate (>70% to 90%) lesions were included in order to allow meaningful exercise levels without safety concerns. The age-matched control group consisted of 15 healthy volunteers (mean age 58±13 years, 5 males, 10 females, P = ns vs. patients). Exclusion criteria were arrhythmia of any kind, a pathological ECG, existing cardiovascular risk factors, current medication affecting myocardial blood flow and/or cardiovascular risk factors and a more than low clinical probability for CAD [16]. All participants were habitual coffee drinkers but refrained from ingesting caffeinated beverages or food for 24 hours before the study.

### Study protocol

With the subject's feet attached to a bicycle ergometer (model 380 B, Siemens-Elema AG, Switzerland) MBF was measured at rest and during supine bicycle exercise-induced hyperemia in all study participants as recently documented [14], [17]. Exercise was started at 25–50 Watts (W), and workload was increased every minute to reach 100% of the predicted value for upright bicycle exercise after 5 minutes. Thereafter, 200 mg of oral caffeine (GlaxoSmithKline, Pittsburgh, PA, USA) - a dose corresponding to two cups of coffee [3] – was administered to each subject and fifty minutes later (at the time of expected serum caffeine peak level) all MBF measurements were repeated. Blood pressure was continuously monitored by a Finapress Monitor (BOC; Inc., Englewood, CO, USA) and recorded every minute at rest, at each exercise level and during recovery. The ECG was monitored continuously and a 12-lead ECG was recorded each minute. Serum caffeine concentration was determined before and 50 minutes after caffeine ingestion.

### Ethics Statement

The local ethics committee approved this study protocol. All subjects gave informed and written consent.

### Image Acquisition

Images were acquired on a Discovery LS PET/CT scanner (GE Healthcare), an integration of an Advance NXi PET scanner with a LightSpeed Plus 4-row helical CT scanner at the PET Centre of the University Hospital Zurich. During the 50-minute break a CT-based attenuation correction was performed [18]. Starting after the background frame, a dose of 500–700 MBq of ^15^O-water was injected as an intravenous bolus over 20 s at an infusion rate of 24 mL/min to assess MBF at rest and after bicycle exercise at baseline conditions and after ingestion of caffeine.

### Image Processing and myocardial perfusion

Images were analyzed with a pixel-wise modelling software developed at our institution (PMOD Technologies Ltd.; Zurich, Switzerland) [20], [24], [26]. Myocardial images were then generated directly from the dynamic ^15^O-water study by factor analysis [19]. After reorienting the factor images along the heart axis to form vertical long-axis, horizontal long-axis, and short-axis-slices, regions of interest were drawn within the left ventricular cavity and myocardium on consecutive image planes and projected onto the dynamic ^15^O-water images to generate blood time-activity-curves. These curves were fitted to a single tissue compartment tracer kinetic model to give values of MBF (mL/min/g) [14], [19], [20], [21]. For the analysis of the controls the segments were grouped to obtain a mean global value for the whole left ventricle. In patients all segments were assigned to either stenosis-dependent (i.e. subtended by a stenotic coronary artery) or remote according to the coronary angiography while blinded for the MBF values by two independent and blinded readers as previously described [14], [22]. Coronary resistance (mmHg/mL/min/g) was calculated as the ratio of mean arterial pressure to MBF at rest and after bicycle stress as reported [17], [19], [20], [21]. Myocardial perfusion reserve (MPR) was defined as the ratio of exercise-induced hyperaemic MBF to resting MBF.

### Statistical analysis

Data are reported as mean values±standard deviation (SD) if not otherwise stated. Demographic data were compared using the chi-square test. Comparisons of hemodynamic and MBF values were performed by ANOVA statistics for repeated measures. When the P-value was <0.05, Sheffe's procedure was applied. For paired comparison of stenotic versus remote segments and unpaired comparison of patients versus age-matched controls, a paired and an unpaired t-test were used, respectively. The number of patients necessary (in each group) was calculated to be fewer than 15, for detecting a minimum clinically relevant difference in hyperaemic MBF of 20% between baseline and caffeine with an alpha of 0.05 and a power (1-beta) of 0.8.

The authors had full access to the data and take responsibility for its integrity. All authors have read and agree to the manuscript as written.
